# Supplementary material for: Unveiling the Bioactive Potential of the Invasive Jellyfish Phyllorhiza punctata Through Integrative Transcriptomic and Proteomic Analyses
Source: Biomolecules. 2025 Aug 4;15(8):1121. doi: 10.3390/biom15081121 (PMC12383608; doi:10.3390/biom15081121)
Supplement: Supplementary file 1 [file biomolecules-15-01121-s001.zip › Suplementary Material/FileS3/PEPFOLD4/XP_065065161_15704/clusters.html]

 PEP-FOLD clusters


Download archive of all models  here   

|  |  |  |
| --- | --- | --- |
| # | file | sOPEP |
| # cluster 1 - Size: 2 |  |  |
|  | XP\_065065161\_15704-model1 | -10.9703 |
|  | XP\_065065161\_15704-model1.1 | -7.87165 |
| # cluster 2 - Size: 1 |  |  |
|  | XP\_065065161\_15704-model2 | -9.87999 |
| # cluster 3 - Size: 3 |  |  |
|  | XP\_065065161\_15704-model3 | -7.68523 |
|  | XP\_065065161\_15704-model3.1 | -4.17738 |
|  | XP\_065065161\_15704-model3.2 | -3.39493 |
| # cluster 4 - Size: 2 |  |  |
|  | XP\_065065161\_15704-model4 | -6.78065 |
|  | XP\_065065161\_15704-model4.1 | -4.58235 |
| # cluster 5 - Size: 3 |  |  |
|  | XP\_065065161\_15704-model5 | -6.37293 |
|  | XP\_065065161\_15704-model5.1 | -5.21094 |
|  | XP\_065065161\_15704-model5.2 | -4.1689 |
| # cluster 6 - Size: 3 |  |  |
|  | XP\_065065161\_15704-model6 | -6.26328 |
|  | XP\_065065161\_15704-model6.1 | -5.91461 |
|  | XP\_065065161\_15704-model6.2 | -5.65795 |
| # cluster 7 - Size: 2 |  |  |
|  | XP\_065065161\_15704-model7 | -6.16453 |
|  | XP\_065065161\_15704-model7.1 | -4.04448 |
| # cluster 8 - Size: 1 |  |  |
|  | XP\_065065161\_15704-model8 | -5.94268 |
| # cluster 9 - Size: 2 |  |  |
|  | XP\_065065161\_15704-model9 | -5.2465 |
|  | XP\_065065161\_15704-model9.1 | -4.64411 |
| # cluster 10 - Size: 2 |  |  |
|  | XP\_065065161\_15704-model10 | -5.19775 |
|  | XP\_065065161\_15704-model10.1 | -5.03666 |
| # cluster 11 - Size: 2 |  |  |
|  | XP\_065065161\_15704-model11 | -5.10254 |
|  | XP\_065065161\_15704-model11.1 | -4.63121 |
| # cluster 12 - Size: 1 |  |  |
|  | XP\_065065161\_15704-model12 | -5.00247 |
| # cluster 13 - Size: 2 |  |  |
|  | XP\_065065161\_15704-model13 | -4.9771 |
|  | XP\_065065161\_15704-model13.1 | -4.32553 |
| # cluster 14 - Size: 1 |  |  |
|  | XP\_065065161\_15704-model14 | -4.9531 |
| # cluster 15 - Size: 4 |  |  |
|  | XP\_065065161\_15704-model15 | -4.95193 |
|  | XP\_065065161\_15704-model15.1 | -4.85155 |
|  | XP\_065065161\_15704-model15.2 | -4.78644 |
|  | XP\_065065161\_15704-model15.3 | -4.71674 |
| # cluster 16 - Size: 1 |  |  |
|  | XP\_065065161\_15704-model16 | -4.88798 |
| # cluster 17 - Size: 2 |  |  |
|  | XP\_065065161\_15704-model17 | -4.80072 |
|  | XP\_065065161\_15704-model17.1 | -4.30127 |
| # cluster 18 - Size: 3 |  |  |
|  | XP\_065065161\_15704-model18 | -4.77715 |
|  | XP\_065065161\_15704-model18.1 | -3.20937 |
|  | XP\_065065161\_15704-model18.2 | -2.33526 |
| # cluster 19 - Size: 2 |  |  |
|  | XP\_065065161\_15704-model19 | -4.76637 |
|  | XP\_065065161\_15704-model19.1 | -3.39053 |
| # cluster 20 - Size: 5 |  |  |
|  | XP\_065065161\_15704-model20 | -4.72706 |
|  | XP\_065065161\_15704-model20.1 | -4.02658 |
|  | XP\_065065161\_15704-model20.2 | -3.95433 |
|  | XP\_065065161\_15704-model20.3 | -3.85002 |
|  | XP\_065065161\_15704-model20.4 | -3.61113 |
| # cluster 21 - Size: 2 |  |  |
|  | XP\_065065161\_15704-model21 | -4.68484 |
|  | XP\_065065161\_15704-model21.1 | -2.68503 |
| # cluster 22 - Size: 3 |  |  |
|  | XP\_065065161\_15704-model22 | -4.66916 |
|  | XP\_065065161\_15704-model22.1 | -2.78353 |
|  | XP\_065065161\_15704-model22.2 | -2.63998 |
| # cluster 23 - Size: 3 |  |  |
|  | XP\_065065161\_15704-model23 | -4.64182 |
|  | XP\_065065161\_15704-model23.1 | -4.52816 |
|  | XP\_065065161\_15704-model23.2 | -3.21252 |
| # cluster 24 - Size: 2 |  |  |
|  | XP\_065065161\_15704-model24 | -4.58926 |
|  | XP\_065065161\_15704-model24.1 | -3.84096 |
| # cluster 25 - Size: 1 |  |  |
|  | XP\_065065161\_15704-model25 | -4.4631 |
| # cluster 26 - Size: 2 |  |  |
|  | XP\_065065161\_15704-model26 | -4.32422 |
|  | XP\_065065161\_15704-model26.1 | -4.25319 |
| # cluster 27 - Size: 2 |  |  |
|  | XP\_065065161\_15704-model27 | -4.28927 |
|  | XP\_065065161\_15704-model27.1 | -3.78018 |
| # cluster 28 - Size: 1 |  |  |
|  | XP\_065065161\_15704-model28 | -4.27538 |
| # cluster 29 - Size: 1 |  |  |
|  | XP\_065065161\_15704-model29 | -4.22442 |
| # cluster 30 - Size: 1 |  |  |
|  | XP\_065065161\_15704-model30 | -4.22279 |
| # cluster 31 - Size: 2 |  |  |
|  | XP\_065065161\_15704-model31 | -4.19224 |
|  | XP\_065065161\_15704-model31.1 | -3.3992 |
| # cluster 32 - Size: 3 |  |  |
|  | XP\_065065161\_15704-model32 | -4.17102 |
|  | XP\_065065161\_15704-model32.1 | -4.06359 |
|  | XP\_065065161\_15704-model32.2 | -3.56139 |
| # cluster 33 - Size: 3 |  |  |
|  | XP\_065065161\_15704-model33 | -4.11018 |
|  | XP\_065065161\_15704-model33.1 | -3.62544 |
|  | XP\_065065161\_15704-model33.2 | -2.44731 |
| # cluster 34 - Size: 3 |  |  |
|  | XP\_065065161\_15704-model34 | -4.08837 |
|  | XP\_065065161\_15704-model34.1 | -3.14622 |
|  | XP\_065065161\_15704-model34.2 | -3.11105 |
| # cluster 35 - Size: 2 |  |  |
|  | XP\_065065161\_15704-model35 | -4.07745 |
|  | XP\_065065161\_15704-model35.1 | -3.39932 |
| # cluster 36 - Size: 2 |  |  |
|  | XP\_065065161\_15704-model36 | -3.84933 |
|  | XP\_065065161\_15704-model36.1 | -3.57324 |
| # cluster 37 - Size: 1 |  |  |
|  | XP\_065065161\_15704-model37 | -3.81733 |
| # cluster 38 - Size: 1 |  |  |
|  | XP\_065065161\_15704-model38 | -3.78039 |
| # cluster 39 - Size: 1 |  |  |
|  | XP\_065065161\_15704-model39 | -3.72409 |
| # cluster 40 - Size: 1 |  |  |
|  | XP\_065065161\_15704-model40 | -3.65088 |
| # cluster 41 - Size: 1 |  |  |
|  | XP\_065065161\_15704-model41 | -3.49387 |
| # cluster 42 - Size: 1 |  |  |
|  | XP\_065065161\_15704-model42 | -3.35979 |
| # cluster 43 - Size: 2 |  |  |
|  | XP\_065065161\_15704-model43 | -3.32497 |
|  | XP\_065065161\_15704-model43.1 | -3.19883 |
| # cluster 44 - Size: 1 |  |  |
|  | XP\_065065161\_15704-model44 | -3.17913 |
| # cluster 45 - Size: 1 |  |  |
|  | XP\_065065161\_15704-model45 | -3.17186 |
| # cluster 46 - Size: 1 |  |  |
|  | XP\_065065161\_15704-model46 | -3.16807 |
| # cluster 47 - Size: 1 |  |  |
|  | XP\_065065161\_15704-model47 | -3.03429 |
| # cluster 48 - Size: 1 |  |  |
|  | XP\_065065161\_15704-model48 | -3.02356 |
| # cluster 49 - Size: 2 |  |  |
|  | XP\_065065161\_15704-model49 | -3.0188 |
|  | XP\_065065161\_15704-model49.1 | -2.79762 |
| # cluster 50 - Size: 1 |  |  |
|  | XP\_065065161\_15704-model50 | -2.86383 |
| # cluster 51 - Size: 1 |  |  |
|  | XP\_065065161\_15704-model51 | -2.79127 |
| # cluster 52 - Size: 1 |  |  |
|  | XP\_065065161\_15704-model52 | -2.78625 |
| # cluster 53 - Size: 1 |  |  |
|  | XP\_065065161\_15704-model53 | -2.76737 |
| # cluster 54 - Size: 1 |  |  |
|  | XP\_065065161\_15704-model54 | -2.68109 |
| # cluster 55 - Size: 2 |  |  |
|  | XP\_065065161\_15704-model55 | -2.65008 |
|  | XP\_065065161\_15704-model55.1 | -2.50582 |
| # cluster 56 - Size: 1 |  |  |
|  | XP\_065065161\_15704-model56 | -2.34078 |
|  |  |  |
